# Nrf2-Activating Natural Compounds in Neurodegenerative Diseases: Targeting Oxidative Stress and Protein Aggregation

**DOI:** 10.3390/ijms27031592

**Published:** 2026-02-05

**Authors:** Lucia Chico, Erika Schirinzi, Linda Balestrini, Maico Polzella, Gabriele Siciliano

**Affiliations:** 1 Laboratori Aliveda S.r.l., 56042 Crespina Lorenzana, Italy; l.balestrini@laboratorialiveda.com (L.B.); maico@aliveda.com (M.P.); 2 Department of Clinical and Experimental Medicine, University of Pisa, 56100 Pisa, Italy; e.schirinzi@gmail.com (E.S.); gabriele.siciliano@unipi.it (G.S.)

**Keywords:** neurodegeneration, Nrf2, protein aggregation, oxidative stress, plant polyphenols, *fungi*, marine organisms

## Abstract

Neurodegenerative diseases (NDs) are among the leading causes of disability and mortality worldwide and are characterized by multifactorial pathogenesis involving interconnected mechanisms, such as oxidative stress, protein misfolding and aggregation, neuroinflammation, and mitochondrial dysfunction. Dysregulation of transcription factors, governing cellular defense responses, particularly nuclear factor erythroid 2–related factor 2 (Nrf2), a key regulator of antioxidant and proteostatic pathways, plays a critical role in neurodegenerative processes. Currently, available pharmacological treatments for NDs are largely symptomatic, as no disease-modifying therapies exist. Natural bioactive compounds have emerged as promising multi-target agents, demonstrating antioxidant, anti-aggregative, and anti-apoptotic properties, frequently mediated through activation of the Nrf2 signaling pathways. These compounds may represent valuable supportive strategies alongside conventional drug treatments, potentially contributing to the modulation of multiple pathogenic mechanisms. This review summarizes key oxidative stress- and protein aggregation-driven mechanisms underlying Alzheimer’s disease, Parkinson’s disease, amyotrophic lateral sclerosis, and Huntington’s disease. It further examines the neuroprotective potential of plant-, fungi-, and marine-derived natural compounds, with particular emphasis on Nrf2 activation. Beyond redox regulation, the broader role of Nrf2 in maintaining proteostasis is discussed. Overall, the review highlights Nrf2-inducing nutraceuticals as promising complementary, multi-target approaches for neuroprotection in NDs.

## 1. Introduction

Neurodegenerative diseases (NDs) are multifactorial disorders characterized by the progressive and selective loss of neurons within the central nervous system (CNS), ultimately leading to cognitive and/or motor impairments [1]. Although each disorder presents distinct clinical and pathological features—such as the involvement of specific brain regions or the accumulation of disease-specific misfolded proteins—NDs also share several converging pathogenic mechanisms. These include protein misfolding and aggregation, chronic neuroinflammation, dysregulation of autophagy, oxidative stress, and mitochondrial dysfunction [2,3].

NDs are debilitating, incurable conditions that profoundly affect the quality of life of the patients and caregivers, requiring increasing long-term medical and social support. To date, no disease-modifying treatments are available. Identifying new therapeutic strategies and neuroprotective compounds capable of preventing or slowing disease progression therefore remains a major challenge. Achieving this goal required an increasingly detailed understanding of the pathophysiological and molecular mechanisms underlying neurodegeneration [4].

Among the pathways of interest, the transcription factor NF-E2-related factor-2 (Nrf2 or Nfe2l2) has gained increasing attention as a central node in the cellular defense network. Beyond its classical role as the master regulator of redox homeostasis, Nrf2 orchestrates a broad cytoprotective program that includes the modulation of detoxification enzymes, mitochondrial function, protein quality control, autophagy, and inflammatory signaling. Because many of these processes are profoundly impaired in NDs, Nrf2 has emerged as a promising biological target for therapeutic exploration [5].

The aim of this review is to provide a comprehensive and critical synthesis of current scientific evidence on natural compounds capable of activating Nrf2 in the context of neurodegeneration. The review focuses on how these bioactive molecules modulate Nrf2 signaling to influence oxidative stress, proteostasis, and protein aggregation—interconnected processes central to major NDs, including Alzheimer’s disease, Parkinson’s disease, Huntington’s diseases, and amyotrophic lateral sclerosis. Mechanistic insights are integrated with preclinical and emerging clinical evidence to discuss the potential contributions of Nrf2-inducing natural compounds in neuroprotective strategies, while also addressing the key limitations, translational challenges, and knowledge gaps that remain in this evolving field.

## 2. Oxidative Stress and Protein Aggregation in NDs: General Aspects

Oxidative stress occurs when the reactive oxygen species (ROS) production exceeds the capacity of antioxidant defenses, perturbing the redox homeostasis. ROS are highly reactive molecules that can damage DNA, lipids, and proteins, impairing their structural integrity and function [6]. The brain is particularly vulnerable to oxidative damage due to its high oxygen consumption, limited antioxidant capacity, and abundance of peroxidizable substrates. Consequently, oxidative stress has long been recognized as one of the major contributors to neurodegeneration [7].

One of the most critical consequences of oxidative damage is protein oxidation, which promotes protein misfolding and aggregation. These events can activate inflammatory pathways and establish a self-perpetuating cycle that further amplifies ROS production, ultimately leading to neuronal death [8]. While toxic protein aggregation represents a shared hallmark across NDs, the biological mechanisms underlying protein inclusion formation remained only partially understood. Among the leading hypotheses are mitochondrial dysfunction and oxidative stress, both implicated in the initiation and progression of aggregation processes.

Overall, growing evidence indicates that oxidative stress and protein aggregation form a tightly interconnected, self-reinforcing cycle that drives neuronal dysfunction and death. This interplay between ROS imbalance and protein pathology provides a conceptual framework common to multiple NDs and sets the stage for the disease-specific mechanisms discussed in the following sections.

### 2.1. Alzheimer’s Disease

Alzheimer’s disease (AD), the most common form of dementia, manifests clinically with progressive cognitive and behavioral decline. Neuropathologically, AD is characterized by diffuse cerebral atrophy and the accumulation of hallmark lesions: intracellular neurofibrillary tangles (NFTs) composed of phosphorylated tau (p-tau) and extracellular senile plaques (SPs) formed by amyloid-β (Aβ_1-42_) peptides [2].

Both pathological features are closely associated with oxidative stress. Aβ toxicity is influenced by peptide concentration, conformation, and intrinsic ROS-generating properties, which can further amplify Aβ production in a feed-forward loop [9]. Redox-active metal ions, including copper and iron, exacerbate this process by promoting free radical formation [10]. Consistently, post-mortem AD brains exhibit elevated oxidative damage markers, including lipid peroxidation products and oxidized nucleic acids, together with reduced antioxidant defenses in both AD and mild cognitive impairment (MCI). Oxidative stress also contributes to tau pathology, as lipid peroxidation-derived aldehydes favor tau hyperphosphorylation and microtubule destabilization [11,12,13].

### 2.2. Parkinson’s Disease

Parkinson’s disease (PD) is the most common movement disorder and is pathologically defined by the selective degeneration of dopaminergic neurons in the *substantia nigra*. Clinically, PD manifests with progressive motor impairment, including bradykinesia, resting tremor, rigidity, and postural instability. The aggregation of the α-synuclein (α-syn) protein, encoded by *SNCA* gene, into Lewy bodies—together with neuronal loss—represents a neuropathological hallmark of the disease [14].

A large body of evidence supports a central role for oxidative stress in PD pathophysiology. Elevated oxidative damage markers and selective depletion of reduced glutathione (GSH) are consistently observed in the *substantia nigra* and peripheral samples of PD patients [12,15].

Oxidative stress actively contributes to PD pathogenesis by promoting α-syn aggregation and the formation of oxidatively modified α-syn species with enhanced neurotoxicity. These processes preferentially affect vulnerable neuronal populations and are further amplified by neuronal hyperactivity, which enhances oxidative and nitrative reactions, α-syn aggregation, and its propagation. Together, these findings support a mechanistic link between oxidative stress, α-syn accumulation, and disease progression [16]. In line with this model, in vitro studies show that α-syn accumulation elevates H_2_O_2_ production, indicating that ROS generation during protein inclusion formation is a key driver of PD-related neurodegeneration [10,17].

Genetic forms of PD linked to mutations in α-syn, parkin, DJ-1, and PINK1 converge on mitochondrial quality control and redox homeostasis, reinforcing the mechanistic link between mitochondrial dysfunction, oxidative stress, and protein aggregation [18].

### 2.3. Huntington’s Disease

Huntington’s disease (HD) is caused by CAG repeats expansion in the huntingtin (*HTT*) gene, resulting in a mutant HTT (mHTT) with an expanded polyglutamine tract [19].

Oxidative stress is considered an early event in HD pathogenesis, preceding and promoting mHtt aggregation. Oxidative modifications—particularly at cysteine residues in the N-terminal fragment—impair mHtt clearance and favor oligomerization [20,21]. Both in vitro and in vivo HD models demonstrate that oxidative stress occurs before aggregate formation, increasing apoptosis [21]. Aggregated mHTT further amplifies ROS production, forming a self-reinforcing cycle. Mitochondrial disfunction is integrated in this process, as mHTT interacts with mitochondrial membranes, disrupts mitochondrial transport and biogenesis, and activates caspases, promoting neurodegeneration [22,23]. Evidence from HD patients shows heterogeneous alterations in oxidative stress biomarkers, with variable levels of protein carbonyls, lipid peroxidation products (MDA, 4-HNE), and oxidized nucleic acids, observed in both brain tissues and plasma [7,24,25].

### 2.4. Amyotrophic Lateral Sclerosis

Amyotrophic lateral sclerosis (ALS) is characterized by the progressive degeneration of upper and lower motor neurons, resulting in weakness of voluntary skeletal muscles involved in limb movement, swallowing, speech, and respiratory function [26]. Neuropathologically, ALS is defined by TAR DNA-binding protein 43 (TDP-43) proteinopathy, involving both loss of nuclear TDP-43 function and formation of cytoplasmic aggregates. Several other protein aggregates are also commonly observed in ALS, including SOD1, fused in sarcoma (FUS), ataxin-2, optineurin, ubiquitilin-2, and the translational product of chromosome 9 open reading frame 72 (C9ORF72) [27].

TDP-43 regulates RNA processing and stability, and its mislocalization triggers both loss- and gain-of-function effects [28]. Nuclear TDP-43 loss disrupts pre-mRNA splicing, fails to repress cryptic exons, and activates retrotransposons, while cytoplasmic aggregates impair RNA granule trafficking, local translation, and mitochondrial function [29]. Chronic and mild oxidative insults promote TDP-43 aggregation and stress granule formation in patient-derived cells, linking ROS to proteinopathy [30].

Similarly, SOD1 mutations (e.g., SOD1-AV4) enhance misfolding and aggregation, which impairs the ubiquitin–proteasome system (UPS) and establishes a vicious cycle in which oxidative stress and protein aggregation reinforce each other [8].

Altered oxidative stress biomarkers have been consistently reported in ALS patients compared with healthy controls [12,31,32]. Oxidative stress can be considered both a cause and a consequence of these proteinopathies. For instance, ROS accumulation triggers TDP-43 aggregation through disulfide-bond formation and cysteine oxidation [33], while TDP-43 aggregates increase neuronal susceptibility to oxidative stress, sustaining a feed-forward cycle that drives motor neuron degeneration [34].

Collectively, these intersecting mechanisms—protein misfolding, impaired RNA processing, UPS dysfunction, and oxidative stress—underlie the onset and progression of ALS.

## 3. Nrf2 Pathway Activation: An Overview

Nrf2, encoded by *NFE2L2* gene, is a master transcription factor coordinating the cellular response to oxidative stress. As a member of the Cap-n-Collar family of basic leucine zipper proteins [35], it binds antioxidant response elements (AREs, 5′-TGAG/CnnnGC-3′) to induce the expression of cytoprotective enzymes, including NQO1, HO-1, SOD1, catalase, GR, GPx, GST, and other detoxifying enzymes [36].

**Figure 1:**
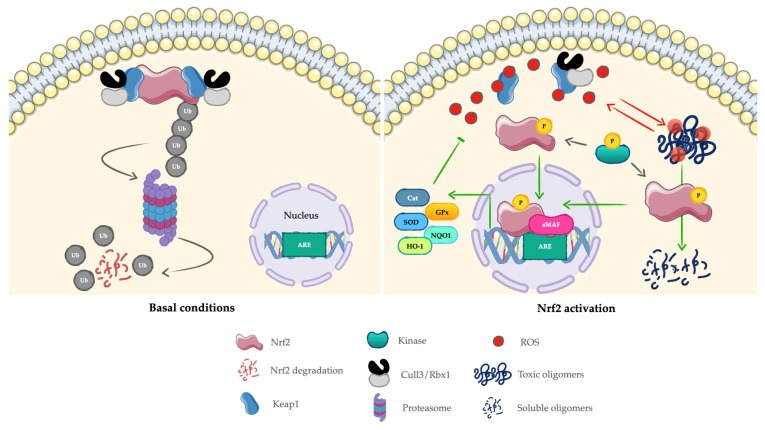
Schematic representation of Nrf2 regulation under basal conditions and following activation in response to oxidative stress and protein misfolding. Green lines indicate the role of Nrf2 in maintaining cellular homeostasis, while red lines indicate oxidative damage. For details, see the main text. Abbreviations: P, phosphate group; Cat, catalase; SOD, superoxide dismutase; GPx: glutathione peroxidase; HO-1, heme oxygenase-1; NQO1: NAD(P)H quinone dehydrogenase 1; ARE, antioxidant response elements, Ub, ubiquitin; ROS: reactive oxygen species.

Under basal conditions, Nrf2 is sequestered in the cytoplasm by Kelch-like ECH-associated protein 1 (keap1), which directs it to the Cullin3/Rbx1 E ligase complex for ubiquitination and proteasomal degradation [37]. Oxidative stress modifies Keap1 cysteines, preventing Nrf2 degradation and allowing its accumulation. Stabilized Nrf2 subsequently translocates to the nucleus, where it dimerizes with small MAF or JUN proteins to bind ARE/EpRE sequences and activate transcription of target genes [38] (Figure 1). Nrf2 activity is modulated by negative feedback loops. Once activated, it can upregulate Keap1/Cullin-3/Rbx-1 components, which in turn restrict Nrf2 accumulation, including nuclear localization. Simultaneously, Nrf2 enhances proteasome activity, establishing an additional layer of autoregulatory control [39].

Beyond its classical role in antioxidant defense, Nrf2 is increasingly recognized as a central regulator of proteostasis—the dynamic maintenance of functional intracellular proteins. Acting as a hub in response to cellular stress, including the accumulation of misfolded or aggregated proteins, Nrf2 coordinates a durable transcriptional response by regulating genes involved in oxidative stress, endoplasmic reticulum homeostasis, proteasome function, and autophagy [40]. Accumulation of misfolded proteins triggers Nrf2 stabilization and nuclear translocation, which enhances the expression of critical components of both proteasomal and lysosomal degradation pathways. This facilitates the clearance of toxic protein aggregates, thereby actively maintaining cellular proteostasis [41].

Tight regulation of this proteostasis network is essential to reduce the toxicity associated with the misfolded or damaged proteins and highlights the potential of Nrf2 as a therapeutic target for diseases characterized by proteostasis imbalance (Figure 1).

Mechanistic evidence supporting a direct role of Nrf2 in proteostasis and the clearance of neuropathological protein aggregates comes primarily from studies enhancing Nrf2 signaling, including genetic approaches and electrophilic compounds such as sulforaphane and tert-butylhydroquinone (tBHQ). These compounds covalently modify critical cysteine residues of Keap1, disrupting the Keap1–Nrf2 interaction. As a result, Nrf2 escapes proteasomal degradation, translocates to the nucleus, and binds to AREs, promoting the transcription of target genes involved in neuroprotection, including molecular chaperones, proteasomal components, and autophagy-related proteins such as p62/SQSTM1 and microtubule-associated protein 1 light chain 3 (LC3), which facilitate the clearance of misfolded and aggregation-prone proteins [42].

In addition, Nrf2 indirectly supports proteostasis by maintaining cellular redox balance and modulating stress-response pathways. By neutralizing ROS, Nrf2 reduces oxidative protein damage, preserves proteome integrity, and enhances heat shock factor 1 (HSF1) activity, upregulating heat shock proteins (HSPs) that assist in proper protein folding and prevent aggregation [41]. Through these direct and indirect mechanisms, Nrf2 integrates proteostatic control with antioxidant defense, establishing a robust protective network that mitigates proteotoxic stress (Table 1).

The following sections examine Nrf2 involvement in NDs, focusing on oxidative stress, proteostasis, and other mechanisms critical for neuronal survival.

### 3.1. Involvement of Nrf2 in NDs

Genetic and epigenetic variability in the Nrf2–Keap1 pathway, including polymorphisms in the NFE2L2 promoter region, can influence Nrf2 activity, antioxidant defenses, xenobiotic metabolism, and susceptibility to NDs. Such interindividual differences may modulate responses to cellular stress and the effectiveness of Nrf2-targeting interventions. Dysregulation of this pathway is increasingly recognized as a central factor in ND pathogenesis.

#### 3.1.1. Nrf2 in AD

Evidence indicates that Nrf2 signaling is impaired in AD, contributing to oxidative stress, impaired protein clearance, and chronic inflammation.

Post-mortem hippocampal tissues show predominant cytoplasmic retention of Nrf2, suggesting defective nuclear translocation and diminished transcriptional activity [43].

Genetic studies also support a role for Nrf2 in AD susceptibility, with protective *NFE2L2* haplotypes enhancing Nrf2 expression, whereas risk haplotypes are associated with earlier onset and faster progression [44].

Experimental models highlight the relevance of Nrf2 for amyloid and tau homeostasis. Nrf2 deficiency APP/PS1 mice increases amyloid precursor protein (APP), Aβ_40_, and Aβ_42_ levels, partly due to loss of Nrf2-mediated repression of β-secretase (BACE1) [45]. Conversely, activation of Nrf2—for example by sulforaphane—reduces BACE1 expression, thereby lowering Aβ production and improving cognitive performance in 3xTg-AD mice [46].

Nrf2 also promotes tau clearance through autophagy, inducing nuclear dot protein (NDP52) and reducing p-tau accumulation, supporting Nrf2-autophagy-mediated degradation of p-tau [47].

Crosstalk with other signaling pathways further modulates Nrf2 activity in AD. For example, the cholinergic anti-inflammatory pathway can activate PI3K/Akt signaling, which inhibits GSK-3β, a kinase that both suppresses Nrf2 and drives tau hyperphosphorylation [36]. Dysregulation of this crosstalk—potentially amplified by toxic Aβ oligomers—can simultaneously impair Nrf2-mediated cytoprotection and promote tau pathology [40].

Overall, Nrf2 supports neuronal survival in AD by enhancing antioxidant defenses, promoting protein clearance, and mitigating proteotoxic stress. Impairments in Nrf2 signaling, due to defective nuclear translocation, genetic susceptibility, GSK-3β–mediated inhibition, and chronic proteotoxic stress, contribute to Aβ and p-tau accumulation, oxidative stress, and neuronal dysfunction.

#### 3.1.2. Nrf2 in PD

Dopaminergic neurons are particularly vulnerable to oxidative stress due to dopamine intrinsic chemical instability. Under physiological conditions, dopamine is sequestrated into synaptic vesicles to avoid cytosolic auto-oxidation [48]. In PD, impaired vesicular storage increases cytosolic dopamine, generating ROS and triggering Nrf2 activation. Nrf2 upregulates *PINK1*, linking redox imbalance to mitochondrial quality control [49]; overexpression of wild-type Nrf2 protects SH-SY5Y cells form oxidative stress, whereas mutated Nrf2 fails to confer such protection [50].

Mutation in *PARK7* (DJ-1), associated with early-onset PD, further underscore this pathway. DJ-1 sequesters Keap1 in the cytosol, stabilizing Nrf2 and enhancing transcriptional activity of antioxidant genes such as NQO1 and GST. DJ-1 deficiency reduces Nrf2-activity and antioxidant defenses [49].

Nrf2 also modulates α-syn proteostasis. By increasing 20S and 26S proteasome expression, Nrf2 facilitates the clearance of α-syn aggregates [51]. Moreover, in neurons with α-syn accumulation, epigenetic de-repression of Nrf2 enhances aggregate clearance, whereas classical Nrf2 activators, such as sulforaphane, are effective mainly in astrocytes, where Nrf2 is active. These findings provide mechanistic evidence that boosting Nrf2 activity can promote the removal of misfolded proteins, supporting the neuroprotective potential of Nrf2-targetig compounds [52].

Iron accumulation in the *substantia nigra*, another PD hallmark, further exacerbates oxidative stress and suppress Nrf2-ARE pathway. Excess ferrous iron downregulates HO-1 through UPS impairment, and in neuronal models inhibits both Nrf2 and HO-1. Despite compensatory HO-1 increases in PD brain, α-syn continues to accumulate, suggesting a dysfunctional antioxidant-proteostatic response [51].

Genetic evidence reinforces this connection: protective *NFE2L2* polymorphisms correlate with delayed PD onset, whereas *KEAP1* variants show limited association [53].

Collectively, these findings highlight Nrf2 as a central node connecting dopamine metabolism, mitochondrial dysfunction, iron dyshomeostasis, and α-syn aggregation. Overall, Nrf2 integrates antioxidant defenses, mitochondrial quality control, and proteostasis, countering dopaminergic neuron vulnerability. Dysregulation at biochemical, cellular, or genetic levels contributes to oxidative stress, iron dyshomeostasis, α-syn aggregation, and PD progression.

#### 3.1.3. Nrf2 in HD

Nrf2 plays a key role in modulating oxidative stress, mitochondrial dysfunction, and protein aggregation in HD, particularly in the striatum [54].

In cellular HD models, including inducible PC12 cells expressing mHTT, early activation of Nrf2-responsive genes such as NOQ1 and GST occurs before aggregate formation, suggesting an early protective response against oxidative stress. After aggregates appear, Nrf2 target expression shows a mixed pattern, reflecting dynamic transcriptional regulation during disease progression [55].

Animal studies further support the neuroprotective role of Nrf2. In 3-nitropropionic acid (3-NP)-induced models, Nrf2 activation is reduced, and knockout mice show increased striatal lesions [56]. Conversely, intrastriatal delivery of Nrf2-overexpressing astrocytes mitigates such lesions, confirming the neuroprotective potential of Nrf2 activation [54,57].

Mechanistic insights highlight HACE1, a regulator of Nrf2 stability and activity. In striatal progenitor cells expressing mHTT, reduced HACE1 correlates with decreased Nrf2 function and increased oxidative stress; restoration of HACE1 rescues Nrf2 activity and redox balance. Notably, HACE1 reduction is observed specifically in the striatum of HD patients, suggesting region-specific vulnerability linked to Nrf2 regulation [58].

Overall, Nrf2-mediated antioxidant and proteostatic mechanisms provide early striatal protection in HD, and modulation of this pathway—directly or via regulators like HACE1—represents a promising neuroprotective strategy.

#### 3.1.4. Nrf2 in ALS

Genetic evidence implicates Nrf2 in ALS pathogenesis. Variants in *NFE2L2* and *KEAP1* have been associated with disease susceptibility and progression. for example, the NFE2L2 haplotype GGGAG correlates with decreased risk, while GAGCAGA, promoting higher Nrf2 expression, delays disease onset [59].

Nrf2 is tightly linked to proteostasis through p62/SQSTM1, which connects misfolded protein clearance with autophagy. Pathogenic SQSTM1 variants highlight the mechanistic interplay between Nrf2, Keap1, and protein homeostasis, suggesting that Nrf2 modulation may counteract ALS pathology [60,61].

Experimental models and patient tissues support Nrf2 involvement in ALS. In TDP-43 (A315T) mice and spinal cord/motor cortex samples from sporadic ALS patients, Nrf2 and its downstream genes (e.g., HO-1) are upregulated, whereas mutant SOD1-expressing cells show reduced Nrf2 activity, indicating protein-specific regulation of antioxidant responses [62].

Nrf2 also contributes to RNA metabolism, a core pathological feature in ALS. Mutations in TARDBP, FUS, and C9orf72 disrupt RNA processing and promote cytoplasmic aggregation of RNA-binding proteins (RBPs), which both reflect proteostasis failure and overload protein quality control systems. In neuronal and glial models, Nrf2 modulates RBP expression, promotes stress granule disassembly, and restores nucleocytoplasmic transport. However, chronic oxidative stress in ALS can impair Nrf2 activation, creating a ROS-resistant state that limits its protective capacity [63].

Collectively, these findings indicate that Nrf2 coordinates antioxidant defense, proteostasis, and RNA metabolism in ALS, and its dysfunction contributes to motoneuron vulnerability. Therapeutic strategies that restore or enhance Nrf2 activity may therefore provide neuroprotection in ALS.

**Figure 2:**
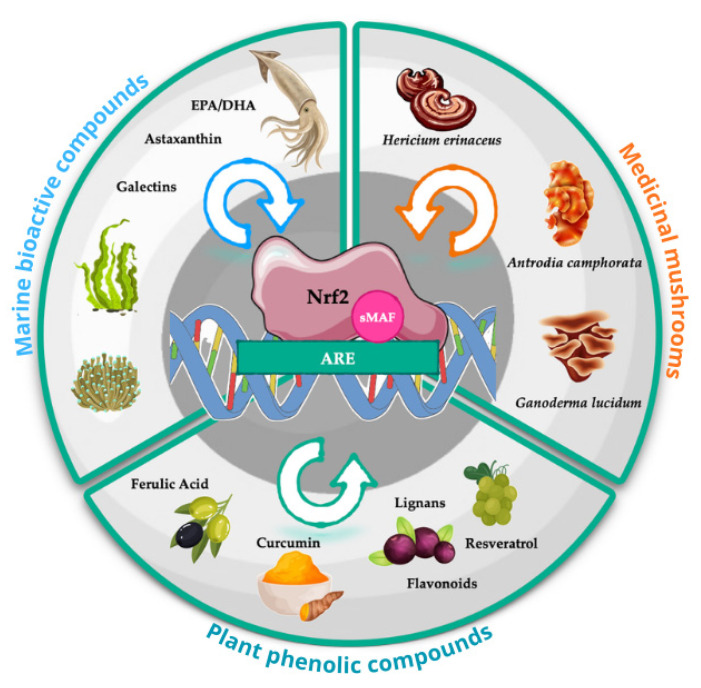
Various plant phenols, medicinal fungi, and marine-derived bioactive compounds can activate Nrf2, promoting its translocation into the nucleus and subsequent activation of Nrf2/ARE pathway. Abbreviations: ARE, antioxidant response elements; EPA/DHA, eicosapentaenoic acid/docosahexaenoic acid.

Despite the pathological alterations affecting Nrf2 signaling in NDs, Nrf2 can still be activated through non-ROS-dependent mechanisms. Both synthetic electrophilic compounds, such as tBQH, and naturally occurring bioactive molecules, including sulforaphane, have been shown to restore Nrf2 activity and mitigate downstream neurotoxic events. Nrf2 activation not only enhances antioxidant defenses but also indirectly supports protein homeostasis, helping to limit the accumulation of misfolded proteins. Naturally derived compounds from plants, fungi, and marine organisms are particularly promising. Their health-promoting effects are largely mediated through the Nrf2/ARE pathway and related antioxidant mechanisms, which counteract the self-perpetuating cycle of oxidative stress and protein aggregation [64] (Figure 2).

The following sections examine the involvement of these compounds as potential Nrf2 activators and assess whether, and to what extent, Nrf2/ARE signaling may contribute to counteracting protein aggregation. Evidence from both preclinical studies and clinical trials will be discussed.

## 4. Plant-Derived Compounds

Plant-derived bioactive molecules have been used in traditional medicine for thousands of years, and modern pharmacology has enabled their rigorous characterization and standardization [63]. Among these, phenolic compounds—including the diverse group of polyphenols—represent the largest class and are commonly categorized into phenolic acid, flavonoids, and non-flavonoid subclasses [64]. These compounds are of particular interest as Nrf2 inducers with potential neuroprotective effects in NDs (Table 2).

### 4.1. Quercetin

Quercetin, a major flavonol, exerts robust neuroprotective effects in preclinical models of NDs. In neuronal cultures, quercetin protects against H_2_O_2_-induced oxidative injury, reducing ROS and pro-inflammatory cytokines production, preventing nuclear and plasma membrane integrity, and modulating apoptotic signaling, largely via Nrf2 activation [61,65]. Specifically, quercetin promotes Nrf2 nuclear translocation and enhances the expression of ARE-dependent genes, without significantly affecting Keap1 expression. However, molecular docking studies suggest a potential interaction between quercetin and the Nrf2-binding site of Keap1, indicating a possible direct modulation of the Keap1-Nrf2 complex [66]. In addition to its antioxidant effects, quercetin destabilizes abnormal protein aggregates and enhances their clearance, suggesting an additional role in the regulation of proteostasis beyond its antioxidant effects [67].

In PD models, quercetin mitigates ferroptosis—a form of iron-dependent lipid peroxidation associated with GSH depletion and mitochondrial alterations—by activating Nrf2. In M17 and PC12 cells, quercetin promotes Nrf2 nuclear translocation and prevents MPP^+^-induced ferroptotic cell death. Notably, Nrf2 knockdown compromises the protective effect, confirming the central role of this pathway in quercetin-mediated neuroprotection [68]. Quercetin directly interferes with a-syn aggregation. In vitro experiments using recombinant a-syn have shown that quercetin (5–100 mM) effectively inhibits fibrillation under aggregation-promoting conditions. Its neuroprotective effects have been attributed to covalent binding with specific amino acids side chains, forming α-syn-quercetin complexes. This increases protein hydrophilicity, disrupts b-sheet assembly, and prevents fibril formation, supporting a direct antiaggregatory mechanism independent of its antioxidant activity [69].

In AD models, quercetin exerts multifaceted neuroprotective effects by modulating oxidative stress, Aβ and tau pathology, and proteostasis-related mechanisms. In Aβ_25-35_-trated PC12 cells, quercetin restores antioxidant enzyme activities (SOD, catalase, GPx), enhances total antioxidant status, reduces lipid peroxidation, and modulates Nrf2 and HO-1 expression, counteracting Aβ-induced oxidative damage [70]. It also reverses Aβ_42_-induced GSK-3β phosphorylation and reduces tau hyperphosphorylation in SH-SY5Y cells and AD-like mice [71]. Quercetin, together with other flavonoids (e.g., 7,8-dihydroxyflavone and apigenin), has been shown to increase the expression of the molecular chaperone HSPB1, involved in the misfolded tau clearance. Since HSPB1 is transcriptionally regulated by HSF1, whose activity can be enhanced by Nrf2, flavonoid-induced HSPB1 upregulation may be at least partially Nrf2-mediated, providing a plausible mechanistic link between Nrf2 activation, improved proteostasis, and mitigation of tau-associated protein aggregation [72].

Importantly, quercetin has been shown to cross the blood–brain barrier (BBB) in preclinical models, enabling direct interaction with CNS targets and exerting neuroprotective effects in vivo. In murine models of aging and dementia, it enhances spatial learning, memory, and overall cognitive performance [71]. In humans, clinical studies results are inconclusive. In a 24-week randomized, double-blind, placebo-controlled trial in healthy older adults, supplementation with quercetin (50 mg/day) prevented cognitive decline, improved depressive symptoms, and increased motivation [73]. Additionally, long-term intervention with quercetin glycoside intake (110 mg/day) was associated with improvements in reaction time and cerebral flow parameters in older adults, although effects on cognition were not consistently significant [74].

Quercetin has been experimentally shown to disassemble preformed fibrils into shorter segments, demonstrating direct anti-aggregation activity [75]. In addition, in ALS cellular models, quercetin (30 µM) and baicalein (30 µM) were tested against both wild-type and A4V-mutant SOD1 in SH-SY5Y neuronal cells. Both flavonoids significantly inhibited fibril formation, blocked fibril elongation, and disaggregated preexisting fibrils, confirming their capacity to interfere with multiple stages of SOD1 aggregation [76]. However, these effects appear largely Nrf2-indipendent, and direct evidence for modulation of Nrf2 signaling by quercetin in ALS is currently lacking.

Similarly, in HD models, quercetin ameliorates mitochondrial dysfunction, antioxidants defenses, and motor and behavioral deficits in 3-NP-treated rats and positively modulates microglial and astrocyte responses [77]. Despite these beneficial outcomes, there is no direct evidence that quercetin exerts its effects in HD via Nrf2 activation.

Taken together, these findings highlight that quercetin’s neuroprotective actions in PD and AD are at least partially mediated through Nrf2-mediated antioxidant and proteostatic mechanisms. In ALS and HD, quercetin appears to act via Nrf2-indipendent pathways affecting mitochondrial function, oxidative stress, and protein aggregation. These observations support the potential of quercetin in neurodegeneration but also underscore the need for disease-specific mechanistic studies to clarify whether and how Nrf2 activation contributes to the mitigation of protein aggregation, particularly in ALS and HD.

### 4.2. Baicalein and Baicalin

Baicalein and baicalin, the major flavones derived from *Scutellaria baicalensis*, exhibit a broad spectrum of neuroprotective activities, including antioxidant and anti-inflammatory effects and inhibition of pathological protein aggregation. Both compounds modulate the interconnected IĸB/NFĸB and Keap1/Nrf2 signaling pathways, acting synergistically to reduce inflammation and oxidative damage while enhancing endogenous antioxidant defense, thereby emerging as promising candidates for NDs [78].

Upon oxidation, baicalin and baicalein form reactive quinone species that are primarily responsible for preventing or inhibiting α-syn fibrillation in vitro, and—of relevance—both flavones can disaggregate pre-formed α-syn fibrils [79,80].

Growing evidence also supports their ability to interfere with Aβ aggregation, suggesting potential utility in modifying or slowing AD progression [81]. Baicalin inhibits Aβ_1–42_ aggregation in a dose- and time-dependent manner and protects SH-SY5Y neurons from Aβ-induced toxicity. Pretreatment with baicalin (0.1–10 μM) significantly increases cell viability following exposure to Aβ_1–42_, with the highest dose (10 μM) improving viability from 57% to 78%. This protective effect is accompanied by reduced H_2_O_2_ production—an oxidative by-product of Aβ aggregation and SPs formation—and mitigation of Aβ-induced oxidative damage [82]. Similarly, baicalein reduces Aβ_25–35_ toxicity in PC12 cells in a dose-dependent fashion, largely through attenuation of oxidative stress [83].

Importantly, while Aβ impairs Nrf2 activation and nuclear translocation, baicalin counteracts this effect by promoting Nrf2 dissociation from Keap1 and subsequent nuclear translocation. Through this mechanism, baicalin enhances endogenous antioxidant defenses by increasing the activity and gene expression of antioxidant and cytoprotective enzymes. Moreover, in vivo, baicalin ameliorates Aβ-induced learning and memory deficits in rats and attenuates hippocampal structural damage [84].

Baicalin also preserves BBB integrity by preventing LPS-induced BBB disruption while activating the Nrf2-mediated antioxidant response, further highlighting its relevance in neuroinflammation [85].

Its neuroprotective effects extend to HD. In the quinolinic acid model, baicalin (10 or 30 mg/kg) restores antioxidant enzyme levels (SOD, catalase, GPx), reduces lipid and protein oxidation, increases BDNF and GDNF expression, and improves motor and behavioral impairments, supporting robust Nrf2-dependent neuroprotective effects [86]. Overall, baicalein and baicalin modulate pathological protein aggregation through both direct, Nrf2-independent mechanisms—such as disassembly of α-syn fibrils—and indirect mechanisms, including the reduction in oxidative damage and the enhancement of Nrf2-mediated antioxidant defenses. These combined antioxidant, anti-inflammatory and anti-fibrillation effects make baicalein and baicalin promising candidates for the modulation of neurodegenerative processes.

### 4.3. Isoflavones

Isoflavones—primarily genistein, daidzein, and glycitein from soy and *Trifolium pratense*—are well-characterized antioxidants capable of directly scavenging free radicals and enhancing endogenous antioxidant defenses. Multiple studies show that isoflavones activate the Nrf2 signaling pathway, thereby reducing lipid peroxidation and increasing the activity of antioxidant enzymes such as SOD, catalase, and GSH-related systems. Their antioxidant and anti-inflammatory effects depend on intact Nrf2 activity, as gene silencing abolishes these protective actions [87,88].

Genistein and glycitein readily cross the BBB, allowing them to exert biological effects within the CNS [87]. Both compounds show anti-amyloidogenic activity in AD models. When incubated with Aβ_1–40_ or Aβ_1–42_ peptides, genistein and glycitein (25–250 μM) inhibit fibril formation, promote destabilization of preformed fibrils, and reduce oligomerization, with glycitein at 250 μM displaying the strongest effect in vitro. These anti-amyloidogenic properties are specifically mediated by direct binding to Aβ monomers, oligomers and fibrils, apparently through mechanisms independent of Nrf2 activation [89]. In vivo studies have supported the beneficial effects of isoflavones: in ovariectomized rat models of AD, dietary isoflavones administration (10–20 g for four weeks) improved working memory and spatial learning [90].

Glycitein also shows neuroprotective activity in cellular models of PD. In human SK-N-SH neuroblastoma cells, it attenuates rotenone-induced oxidative stress and increases cell viability in a concentration-dependent manner (2.5–20 μM), accompanied by increased SOD, catalase, and GSH activity [91]. Similar protective effects are observed in primary mesencephalic cultures exposed to rotenone or to an adenovirus encoding the familial PD-associated A53T α-syn mutant. Soy-derived isoflavones –including genistein, glycitein, daidzein (100 nM), and its metabolite equol (50 nM)—prevent dopaminergic neuron loss and neurite retraction under these PD-relevant insults. *T. pratense* extract, but not soy isoflavones, further enhances Nrf2 mRNA expression and activates the Nrf2–ARE pathway in cortical astrocytes, while inhibiting UPS activity. In vivo, the soy-derived isoflavone mixture (5–20 mg/kg for 39 days) ameliorates motor deficits in a 6-OHDA-induced PD model [92].

Isoflavones have also shown promise in ALS models. In SOD1^G93A^ mice, genistein (16 mg/kg twice daily) induces autophagy, facilitating the clearance of misfolded and aggregated proteins in motor neurons. Treated mice exhibit delayed symptom progression, reduced motor impairment, and prolonged survival—particularly in males [93].

Despite compelling preclinical evidence, clinical translation remains limited. A randomized clinical trial (NCT00205179) testing soy isoflavones (100 mg/day for 6 months) in AD patients found no significant improvement in cognitive performance. However, interindividual variability in isoflavone metabolism—especially the capacity to convert daidzein into equol—is increasingly recognized as key determinants of therapeutic response [94]. Since equol-producers display higher circulating levels of bioactive metabolites and enhanced antioxidant and anti-inflammatory responses, these metabolic differences may critically influence the degree of Nrf2 activation in vivo. Future studies integrating metabolic phenotyping may therefore be necessary to fully clarify the neuroprotective potential of isoflavones in humans [95].

### 4.4. Phenolic Acids

Phenolic acids—particularly ferulic acid (FA), caffeic acid, and caffeic acid phenethyl ester (CAPE)—exert potent neuroprotective actions largely mediated by activation of the Keap1/Nrf2/ARE pathway. Owing to their chemical structure, these compounds act as strong antioxidants that enhance endogenous defense systems, reduce ROS accumulation, and attenuate neuroinflammatory cascades.

FA activates the Keap1/Nrf2/HO-1 axis, increasing antioxidant capacity and limiting ROS-mediated damage [96]. Beyond its redox-regulating properties, FA directly interferes with amyloidogenesis: in vitro, it destabilizes Aβ fibrils and prevents the formation of new aggregates in a dose-dependent manner [97]. In vivo, chronic FA administration protects mice from A_β1-42_-indiced learning and memory deficits, substantially reducing plaques-associated oxidative stress [98].

FA-mediate Nrf2 activation is also relevant in PD models. FA prevents MPP^+^/MPTP-induced oxidative injury by lowering MDA and lipid hydroperoxides while restoring GSH homeostasis. Mechanistically, FA activates ERK1/2-Nrf2-ARE pathway and promotes de novo Nrf2 synthesis, leading to increased nuclear Nrf2 accumulation [99]. The functional importance of Nrf2 is demonstrated in vivo: FA improves motor deficits in wild-type and α-syn-knockout mice treated with MPTP, but not in Nrf2 knockout mice, confirming that its neuroprotective effect is Nrf2-dependent. In *C. elegans* PD models, FA reduces α-syn accumulation, preserves dopaminergic neurons, lowers ROS production, and improves motor behavior [100].

Other phenolic acids have similar Nrf2-driven neuroprotection. Caffeic acid and FA protect cerebellar granule neurons from oxidative, nitrosative, and glutamate-induces excitotoxic stress [101], suggesting relevance for ALS-related pathways. CAPE, a potent Nrf2 inducer, has shown robust efficacy across multiple neurodegeneration models. In SOD1^G93A^ mice, CAPE increases lifespan and motoneuronal survival while reducing microglial and astrocytic activation [102]. In HD models, CAPE (30 mg/kg) decreases 3-NP-induced striatal damage, improves motor performance, and reduces mortality by nearly 30% [103]. Notably, CAPE is described as a pathology-targeted Nrf2 activator, preferentially triggering Nrf2 signaling under oxidative stress conditions [104].

Phenolic acids activate Nrf2 and exert neuroprotective effects, including the reduction in protein aggregation. However, current evidence does not conclusively demonstrate that their anti-aggregation properties are directly mediated by Nrf2. While Nrf2 likely contributes to overall cytoprotection and the maintenance of proteostasis, a causal link between Nrf2 activation and reduced protein aggregation for these specific compounds remains unproven.

### 4.5. Resveratrol and Grape-Derived Polyphenols

Resveratrol and other grape-derived polyphenols can cross the BBB, exerting neuroprotective effects. Well-known for activating Sirtuin 1, resveratrol enhances also Nrf2 expression and activity, upregulating antioxidant and phase II enzymes, reducing ROS, Fe^2+^ and MDA, and increasing GSH in rotenone-treated microglial BV-2 cells [105,106].

In PD models, resveratrol alleviates L-DOPA-induced dyskinesia, protects dopaminergic neurons, and reduces microglial/astroglial activation, while co-administration with L-DOPA maintains therapeutic efficacy [107,108]. Resveratrol and grape seed polyphenolic extracts (GSPEs) also prevent α-syn aggregation, restore motor function, and mitigate oxidative stress in MPTP- and maneb-paraquat-induced models.

In AD, resveratrol inhibits Aβ_42_ fibril formation and reduces Aβ-induced oxidative damage in vitro [109]. GSPEs mitigate tau hyperphosphorylation, dissociate tau aggregates, and improve cognitive function in AD mouse models [110,111]. Clinical trials in mild-to-moderate AD indicate that long-term resveratrol intake (52 weeks, dose escalation 500–2000 mg/day) stabilizes CSF and plasma Aβ_40_ levels, with similar non-significant trends for Aβ_42_, tau and p-tau levels. Resveratrol also lowers CSF markers of neuroinflammation (TREM2), tissue injury (metalloprotease 9) and impaired autophagy (cathepsin D), though cognitive outcomes (MMSE, CDR, ADAS-Cog, NPI) remained unchanged [112,113]. A second randomized trial testing daily grape intake (72 g/day for 6 months) shows improved regional brain metabolism—particularly in the posterior cingulate and temporal cortex—without measurable cognitive gains. Nevertheless, metabolic activity in specific cortical regions correlated with better attention and working memory, suggesting a functionally neuroprotective effect of grape-derived polyphenols [114].

In HD, resveratrol shows mixed results: short-term administration improves motor function and mitochondrial activity in YAC128 and N171-82Q mice [115], whereas other studies report no significant benefits [116]. Clinical trials assessing resveratrol effects on caudate volume in early HD patients have been performed (NCT02336633), but no results are currently available.

In ALS, resveratrol demonstrated partial neuroprotection in SOD1G93A mice, delaying disease onset, extending lifespan, and preserving motor neuron function in some studies [117], while other trials reported minimal effects on survival but improvements in motor performance and muscle strength [118,119]. It also protects rat cortical motoneurons against ALS patient CSF-induced toxicity, unlike riluzole [120]. Clinical evaluation of combined resveratrol and curcumin supplementation in ALS patients is underway (results not yet available, NCT04654689).

Overall, resveratrol ang grape-derived polyphenols exert pleiotropic neuroprotective effects, including enhancement of antioxidant defenses and modulation of proteostasis through both direct and indirect mechanisms. Directly, Nrf2/ARE activation upregulates antioxidant and cytoprotective enzymes and may promote expression of autophagy-related genes, contributing to the clearance of misfolded proteins. Indirectly, resveratrol reduces ROS, activates SIRT1, and attenuates neuroinflammation, thereby creating a cellular environment conducive to proteostasis and limiting protein misfolding and aggregation.

Although Nrf2 contributes to the cytoprotective and proteostatic effects of resveratrol, a causal link between Nrf2 activation and direct anti-aggregation activity remains unproven, and the observed neuroprotection likely reflects the combined action of multiple convergent mechanisms.

### 4.6. Curcumin

Curcumin, a hydrophobic polyphenolic compound extracted from *Curcuma longa* L., demonstrates well-established antioxidant and anti-inflammatory properties and represents one of the most extensively studied natural Nrf2 activators in neurodegeneration [121].

At the molecular level, curcumin directly interacts with Keap1 by binding to the Cys151 residue, which is recognized as a critical redox-sensitive site for Keap1 modification. This interaction facilitates the dissociation of the Keap1–Nrf2 complex, promoting Nrf2 stabilization and nuclear translocation. Consistently, the α,β-unsaturated carbonyl moiety of curcumin appears to be essential for its electrophilic interaction with Keap1 and for the sustained activation of Nrf2 signaling [122]. As a consequence, curcumin activates the Nrf2/ARE pathway, upregulating phase II detoxification and antioxidant enzymes, while protecting against apoptosis [123]. Importantly, curcumin exhibits dose-dependent effects: low-to-moderate doses reduce oxidative stress markers (e.g., MDA, ROS) and promote Nrf2 nuclear translocation, whereas high doses paradoxically increase oxidative stress [123]. In astrocytes and neurons, curcumin induces HO-1 expression in an Nrf2-dependent manner, with optimal response at 25 µM and 15 µM, respectively [124].

Curcumin therapeutic potential extends beyond antioxidant mechanisms. Its structural features—particularly the phenolic hydroxyl and phenyl methoxy groups—confer anti-amyloidogenic activity, in a multitarget profile that makes curcumin attractive for proteinophaties. In APP/PS1 mice, chronic curcumin administration (50–200 mg/kg for 3 months) improves learning in a dose-dependent and reduces hippocampal Aβ deposits and SPs formation [125]. Mechanistically, curcumin binds to Aβ_1–42_ fibrils and inhibits the deposition of Aβ_1–40_ and Aβ_1–42_ as oligomers, preventing their conversion to fibrillar forms. Rather than directly blocking fibril formation, curcumin appears to shift the aggregation pathway toward soluble oligomers and prefibrillar aggregates with reduced neurotoxic properties, thereby preventing Aβ_1–40_ toxicity [126]. In addition, curcumin targets tau aggregation. In vitro studies demonstrate that curcumin inhibits hyperphosphorylated tau accumulation and disintegrates existing tau fibers. In tau transgenic animals, curcumin suppresses GSK-3β activity, reducing tau hyperphosphorylated oligomerization, and dimer formation [126].

In PD models, chronic oral curcumin (50–200 mg/kg for 3 weeks) improves locomotor activity and motor coordination while reducing MDA and enhancing SOD, catalase, and GSH levels [127]. These effects appear mediated by direct Nrf2 phosphorylation and activation [128]. Notably, prophylactic curcumin treatment proves more effective than post-exposure, as demonstrated in LRRK2-mutation fibroblasts exposed to paraquat, where pretreatment preserves mitochondrial function [129].

In MPTP-induced PD mice, curcumin combined with levodopa significantly reduced α-syn accumulation and oxidative stress while enhancing neuronal survival compared to monotherapies, effects amplified by lactoferrin-based delivery systems [130].

In a rotenone-induced PD mouse model, curcumin (80 mg/kg for 35 days) activated Nrf2 and restored autophagy via the p62-Keap1-Nrf2 pathway. This led to enhanced clearance of misfolded a-syn by increasing the LC3-II expression, providing a mechanistic example of how Nrf2 activation by curcumin can mediate neuroprotection, mitigating protein aggregation directly through autophagy [131].

Curcumin anti-aggregation properties extend to HD models. In CAG140 knock-in mice, lifelong dietary curcumin reduces striatal mHTT aggregates, increases dopaminergic signaling (DARPP-32, D1 receptor mRNA), and improves motor behavior [132]. Mechanistically, curcumin interacts with the mHtt, delaying aggregation and altering fibril morphology: filaments become narrower with reduced bundling propensity. Although aggregation is not completely blocked, curcumin mitigates mHTT-induced mitochondrial stress. Current evidence indicates that curcumin’s narrowing of mHtt fibrils and reduced bundling arise from direct biophysical modulation of HttEx1 aggregation, not primarily from Nrf2-mediated transcriptional responses, although Nrf2 may contribute to separate downstream cytoprotective effects [133].

Monocarbonyl dimethoxy curcumin (compound C) reduces oxidative damage in NSC34 motor neurons expressing Q331K TDP-43 mutation, decreasing MDA and LDH levels while inhibiting TDP-43 fragmentation and TDP-25 generation. In C57BL/6 mice, compound C induces hippocampal HO-1 expression, suggesting protection against TDP-43 toxicity [134]. Curcumin modulates multiple ALS-associated protein aggregates. In THP-1 cells, curcumin inhibits SOD1 fibril formation and reduces aggregate size in soluble fraction [135]. Interestingly, structural studies reveal that curcumin binds to hydrophobic amino acids within preformed SOD1 fibrils and stabilizes rather than destabilizes these structures [75]. Beyond SOD1, curcumin potentially suppresses FUS droplet formation and stress granule aggregation. Binding studies demonstrate hydrophobic interactions that reduce FUS β-sheet content and prevent pyruvate kinase sequestration, thereby restoring glycolytic metabolisms and ATP production [136]. For ALS related SOD1 and FUS, curcumin’s inhibition of fibril formation, droplet formation, and stress granule aggregation is best explained by direct biophysical interactions with the proteins/condensates, with no current evidence that these specific aggregation effects are mediated by Nrf2, though Nrf2 may contribute to broader cytoprotective outcomes.

#### Clinical Trials

Despite robust preclinical data, clinical trials show variable results, largely attributable to curcumin’s poor bioavailability and limited BBB penetration. Although curcumin exhibits a favorable safety profile, these pharmacokinetic limitations substantially restrict its therapeutic potential and represent major challenges for effective CNS delivery [137].

Consistent with these limitations, clinical trials investigating curcumin in neurodegenerative diseases have reported heterogeneous outcomes. In AD, a 6-month randomized trial combining curcumin (1 or 4 g/day) with ginkgo extract (120 mg/day) failed to improve cognitive function (MMSE scores) or modify Aβ levels, despite increasing vitamin E levels (NCT00164749) [138]. Notably, no bioavailability-enhancing formulation was used.

In ALS, three trials evaluated curcumin as add-on to riluzole using improved delivery systems. A monocomponent nanocurcumin preparation (80 mg/day for 12 months) improved survival probability but not functional outcomes (ALSFRS-R score, muscle strength, and compound muscle action potential) [139]. A more complex formulation—combining curcumin with *Camelia sinensis*, *Centella*, *Ashwagandha*, *Bacopa*, and Coenzyme Q10 and delivered via lactoferrin-based carrier—showed beneficial effects on multiple parameters. At 600 mg/day for 6 months, it produced a modest attenuation of functional decline (ALSFRS-R) together with improvements in aerobic metabolism and oxidative stress markers [32]. At the higher curcumin dose of 1500 mg/day for 6 months, the same formulation yielded more pronounced responses, including reduced protein oxidation, increased non-enzymatic antioxidant capacity (at both 3 and 6 months), decreased IL-6 levels (at both 3 and 6 months), and stabilization of TNFα, which worsened in the placebo group [140].

### 4.7. Emerging Compounds Targeting Nrf2

Recent preclinical evidence has identified other phenolic compounds as promising Nrf2 activators with neuroprotective potential, particularly gallic acid (GA) and lignans.

GA, a polyphenol abundant in walnuts, tea, and grapes, demonstrates robust antioxidant and anti-inflammatory properties through Nrf2-dependent mechanisms. In of chronic sleep deprivation models—which recapitulate key features on neurodegeneration including oxidative stress, neuroinflammation, and cognitive decline—GA administration restores memory function, normalizes antioxidant capacity (TAC, SOD), reduces lipid peroxidation (MDA), and proinflammatory cytokines (IL-1β, IL-6, and TNF-α). These effects correlate with upregulation of Nrf2, HO-1, and NQO1, alongside suppression of NF-κB signaling [141]. Complementary studies in PC12 cells show that GA protects against tBHP-induced oxidative injury by enhancing Nrf2/HO-1 activation and ROS reduction, while in vivo models of spinal cord ischemia–reperfusion injury demonstrate improved neurological outcomes and decreased oxidative-inflammatory markers [142].

Lignans, commonly found in cereals, nuts, flaxseed, vegetables, fruits, and various beverages, exert antioxidant and anti-inflammatory effects through modulation of mitophagy, apoptosis, and transcriptional regulation [143]. Accumulating evidence suggests that multiple lignans activate Nrf2, often via AMPK-dependent pathways. Notably, *Eucommia ulmoides* extracts activate the PI3K/AKT/GSK-3β/Nrf2 axis in H_2_O_2_-treated PC12 cells and in models of tauopathy [144]. Magnolol, a representative lignan, reduces oxidative stress, suppresses NLRP3 inflammasome activation, and upregulates Nrf2/HO-1 signaling, contributing to antidepressant and anti-inflammatory effects in vivo [145]. Besides Nrf2 modulation, certain lignans exhibit direct anti-amyloidogenic activity. Compounds from *Styrax japonica* stem bark—including styraxlignolide A, masutakeside I, and egonol—potentially inhibit BACE1, limiting Aβ production. Styraxlignolide A showed the strongest activity with minimal cytotoxicity, while egonol additionally prevented Aβ42 aggregation and demonstrated BBB permeability [146].

Together, GA and lignans represent a promising class of naturally occurring Nrf2 activators that address multiple pathological hallmarks of neurodegeneration: oxidative stress, inflammation, and protein aggregation. Their capacity to enhance endogenous antioxidant defense while modulating amyloidogenic pathways makes them attractive candidates for further investigation. However, current evidence remains limited to preclinical models, and clinical validation is needed to establish therapeutic potential in NDs.

## 5. Edible Fungi Able to Induce Nrf2

Medicinal fungi produce diverse bioactive metabolites—polysaccharides, polyphenols, terpenoids, alkaloids, and lectins—that show antioxidant and anti-inflammatory effects, mitigating neuronal damage and supporting proteostasis in neurodegenerative conditions [147]. Among these, *Antrodia*, *Ganoderma*, and *Hericium* have attracted particular interest, for their ability to activate Nrf2 and mediate neuroprotection.

### 5.1. Antrodia camphorata

Bioactive compounds from *Antrodia camphorate,* including antroquinonol and polysaccharides, activate Nrf2 and downstream antioxidant and cytoprotective pathways. [148]. In APP transgenic mice, early treatment (34.2 mg/kg/day, starting at 3 months of age) reduced hippocampal total Aβ and Aβ_42_ levels, increased Nrf2 expression, decreased histone deacetylase 2, and improved memory and spatial learning [149].

In PD models, polysaccharides and mycelium extracts restored dopaminergic neurons, reduced α-syn accumulation, and downregulated NLRP3 inflammasome. These effects were associated with Nrf2 activation, contributing to both antioxidant defense and proteostasis regulation [150,151]. Treatment with *Antrodia camphorata* further modulates antioxidant enzymes including Mn-SOD and HO-1, enhancing neuronal resistance to oxidative stress and inflammation. Notably, *A. camphorata* administration decreased the number of α-syn-positive neurons and reduced phosphorylated α-syn levels, supporting its Nrf2-mediated neuroprotective and proteostasis-promoting effects [152].

### 5.2. Ganoderma lucidum

*Ganoderma lucidum* (Reishi) produces bioactive compounds, including triterpenoids (GLTs) and ganoderic acid (GA), which active Nrf2 and downstream cytoprotective enzymes. GLTs attenuate cognitive impairment in APP/PS1 mice, decreasing oxidative stress markers (MDA, lactic dehydrogenase), apoptosis, and modulating antioxidant defenses such as SOD, highlighting their contribution to proteostasis and neuronal survival [153]. GA-C2 specifically enhances NGF expression in primary astrocytes, supports mitochondrial biogenesis, and restores behavior and neuronal integrity in 3-NP-induced HD models [154]. Ethanol extracts of protect myoblast cultures against H_2_O_2_-induced oxidative damage via Nrf2 expression, nuclear translocation, and HO-1 induction, reinforcing cellular defense mechanisms [155].

GLPS, a polysaccharide from *G. lucidum*, reduces p-tau and Aβ levels in 5× FAD mice, likely via downregulation of APP and BACE1, and improves cognitive performance. AD mice exhibited decreased Nrf2 and downstream antioxidant enzymes (HO-1, NQO1, SOD2), which were restored by GLPS, suggesting neuroprotective and antioxidative effects mediated, at least partly, through the Nrf2/antioxidant enzyme pathway. GLPS also mitigated mitochondrial fragmentation, thereby supporting neuronal homeostasis [142].

In preclinical models, GA protects neurons, reducing hippocampal neurodegeneration, preserving mitochondrial function, and up-regulating survival-relative proteins including BDNF and Nrf2, thereby maintaining spatial and non-spatial memory [156]. GA-D delays senescence in human mesenchymal stem cells via the PERK/Nrf2 pathway: PERK phosphorylation promotes Nrf2 nuclear translocation and upregulates antioxidant genes (PRDX3, HO-1, NQO1), reducing ROS accumulation and alleviating cell cycle arrest, further demonstrating the capacity of *G. lucidum* derivatives to enhance cellular resilience [157].

### 5.3. Hericium erinaceus

*Hericium erinaceus* has been investigated for its neuroprotective properties, with its bioactive compounds—particularly erinacines and hericenones—modulating oxidative stress and activating cytoprotective pathways, including the Nrf2/ARE signaling axis [158,159]. Ethanolic basidiocarp extracts (HE-ETH) increase neuronal viability, reduce ROS, enhance catalase and GSH levels, and improve mitochondrial function [159]. Erinacine A and C inhibit Keap1, promoting Nrf2 nuclear translocation and HO-1 induction, thereby supporting proteostasis and neuronal resistance to oxidative stress [160,161].

In vivo HE-ETH attenuates cerebral Aβ deposition and promotes neurogenesis in APP/PS1 mice [162], and improves oxidative stress parameters, dopaminergic neuron survival, and motor performance in rotenone-induced PD mice [147].

Clinical trials report cognitive benefits of *H. erinaceus*. In MCI patients, 16-week supplementation (720 mg/day) improved cognitive scores (HDS-R), although effects reversed after 4 weeks of washout [163]. Similar benefits were observed after 12-week supplementation with fruiting body extracts (3.2 g/day) assessed by MMSE [164]. In mild AD patients, thrice-daily oral erinacine A-enriched *H. erinaceus* mycelia, (350 mg/capsule, 5 mg/g erinacine A) improved MMSE, and IADL scores and stabilized biomarkers including calcium, albumin, ApoE4, and Aβ_1–40_, compared to placebo [165]. While these clinical studies do not directly confirm Nrf2 activation, they support the translational potential of *H. erinaceus* as a neuroprotective agent.

Bioactive compounds from edible and medicinal fungi exert neuroprotection primarily through Nrf2-mediated antioxidant and cytoprotective pathways, mitigating oxidative stress, supporting proteostasis, and reducing pathological protein accumulation in preclinical models of PD and AD. These Nrf2-dependent mechanisms provide a mechanistic rationale for the cognitive and neuroprotective benefits observed in preliminary clinical studies.

## 6. Compounds of Marine Derivation

Marine organisms, including algae, fish, shellfish, and microalgae, produce bioactive compounds such as ω-3 polyunsaturated fatty acids (ω-3 PUFAs), particularly eicosapentaenoic acid (EPA) and docosahexaenoic acid (DHA), carotenoids, and peptides. These molecules provide strong antioxidant and neuroprotective effects, protecting neuronal cells from oxidative damage, preserving membrane integrity, and in some cases enhancing the stability or bioavailability of co-administered bioactives [166,167].

Xyloketal B, isolated from the mangrove fungus *Xylaria* sp., modulates apoptotic proteins in neurons and microglia during ischemia, reducing oxidative damage. Among its mechanisms, Xyloketal B activates the Nrf2/ARE pathway, inducing HO-1 expression of the antioxidant stress protein [168]. This suggests potential utility in AD, where brain ischemia is implicated in pathogenesis.

Galectins are β-galactosidase-binding proteins found in fish such as Atlantic salmon (*Salmo salar*) and flounders, involved in neuronal regeneration and inflammatory modulation [169]. Among them, Galectin 1—expressed in the CNS—exhibits context-dependent effects. In SOS1^G93A^ mice, Galectin 1 accumulates in neurofilamentous lesions before symptom onset, correlating with early axonal degeneration, while astrocytic Galectin 1 may contribute to axonal degeneration during symptomatic stages, indicating a non-protective role in ALS [170]. In contrast, Galectin 1 demonstrates neuroprotective effects in other experimental models. In SH-SY5Y cells exposed to MPP^+^, Galectin 1 reduced ROS production, prevented apoptosis, and protected against DNA damage, effects abolished by Nrf2 knockdown, suggesting Nrf2-dependent neuroprotection [171]. Galectin 1 protective actions are partly mediated through Nrf2 activation, potentially linked to AMPK phosphorylation, whereas Galectin 3 exhibits pro-inflammatory properties [172,173]. Collectively, these findings suggest that Gal-1 effects are context- and disease-specific, providing protection in certain oxidative or inflammatory conditions, but not in others, as ALS.

DHA and EPA, found in fish roe, shrimp and shellfish, activate Nrf2 signaling and suppress LPS-induced inflammation in mice [174]. Adequate brain DHA levels support neuronal transmission, plasticity, glucose uptake, and dopamine storage. By activating Keap1/Nrf2 pathway, these ω-3 PUFAs also promote neuroprotection and contribute to metabolic homeostasis via mitochondrial regulation and axonal transport [175]. DHA/EPA levels are reduced in AD and PD patients. Clinical studies show mixed results: in moderate AD, 6-month supplementation (430 mg DHA plus 150 mg EPA) exerted immunomodulatory effects but did not significantly affect oxidative stress [176]. In ApoE-E4 mice, lysophosphatidylcholine-DHA supplementation restored hippocampal DHA levels and conferred neuroprotection [177], with anti-inflammatory effects reported in PD models and human studies [178].

Astaxanthin, a carotenoid present in shrimp, salmon, and crab, exhibits strong antioxidant and neuroprotective effects through Nrf2–ARE activation and suppression of NF-κB-mediated inflammation [179]. In tauopathy models, it protects cells expressing ΔK280 mutant tau by preventing viability loss and apoptosis and by normalizing caspase-3 and Bcl-2 activation [180]. Astaxanthin also inhibits inflammation and ferroptosis by regulating Keap1-Nrf2/HO-1 axis [181]. In vivo, treatment of APP^NL-G-F^ mice reduced amyloid deposition, decreased phospho-tau, and enhanced microglia activation around plaques [182].

While the direct role of marine-derived compounds in proteostasis and protein misfolding remains unproven, their ability to activate Nrf2 may indirectly support protein homeostasis. Nrf2 induction can engage protective pathways such as autophagy and the reduction in oxidative stress and neuroinflammation, creating a cellular environment less permissive to protein aggregation and neurodegeneration.

Overall, despite their neuroprotective potential, not all natural compounds discussed have been evaluated in clinical trials, and some have yielded inconclusive results. For ease of comparison, the main clinical trials mentioned in the text are summarized in Table 3.

## 7. Strategies to Enhance Bioavailability and BBB Penetration of Natural Compounds

The clinical translation of natural compounds is frequently hampered by poor aqueous solubility, chemical instability, rapid metabolism, and limited oral bioavailability, with BBB impermeability representing an additional major obstacle in CNS disorders. Nanotechnology-based delivery systems address these challenges by improving solubility, protecting compounds from degradation, enhancing bioavailability, and facilitating BBB penetration through adsorptive-mediated or receptor-mediated transcytosis [183].

Lactoferrin-based nanoparticles exemplify successful CNS-targeted systems, exploiting lactoferrin’s high affinity for receptors on cerebral endothelial cells. Allied to levodopa and curcumin, these formulations enhanced brain accumulation and, in preclinical models, reduced Aβ plaques and α-syn aggregates, while improving neuronal survival [130,184]. Various nanoformulations—polymeric nanoparticles, solid lipid nanoparticles (SLN), liposomes, micelles, polymeric conjugates, and nanoemulsions—have been developed for CNS delivery of natural compounds.

Curcumin-encapsulated exosomes demonstrate high bioavailability, safety, and immunomodulatory capacity. In AD models, they cross the BBB, accumulate in neurons, and inhibit tau hyperphosphorylation via AKT/GSK3β activation. Curcumin derivatives with lipid ligands (phosphatidic acid, cardiolipin, GM1 ganglioside) or nanoliposomal formulations inhibit fibrillar and oligomeric Aβ formation in vitro [185], while chitosan nanocapsules increase oral bioavailability nine-fold with BBB permeability [186].

Quercetin nanoformulations include MQNPN, Qc@SNPs-MB, and polymer-based carriers. In AD models, MQNPN reduced hippocampal damage, decreased MAPT and APP expression, and enhanced antioxidant activity. Ultrasound-assisted Qc@SNPs-MB delivery in APP/PS1 mice reduced Aβ plaques, increased neuronal survival, and improved cognition. In HD models, nanoquercetin inhibited mutant huntingtin aggregation at lower concentrations than free quercetin [187].

Resveratrol crosses the BBB naturally but undergoes rapid metabolism to glucuronide and sulfate conjugates, limiting brain concentrations. Nanoformulations (SLN, PLGA-based carriers, galactosylated PLGA, polymeric micelles) improve solubility, protect against metabolism, and enhance CNS delivery. These systems significantly increased oral bioavailability, systemic half-life, and brain accumulation. Resveratrol-loaded micelles protected PC12 cells against Aβ toxicity where free resveratrol failed. Nanoformulated resveratrol achieved neuroprotection at five-fold lower doses, while SLN-TPGS formulations increased AUC 11-fold and half-life nine-fold [188,189].

Intranasal nanoformulated DHA improved well-being and spatial memory in J20 mice, reducing amyloid burden, oxidative stress, and neuroinflammation via GSK3β inhibition [190]. Cyclodextrin-based nanosponges (CD-NSs) for ω-3 PUFAs enabled enhanced CNS targeting. In AD and PD models, ω-3-loaded CD-NSs outperformed free fatty acids, improving cognition and reducing neuroinflammation (decreased TNF-α, IL-1β, IL-6 in brain tissue and CSF). These effects accompanied reduced neuronal degeneration in cortex, substantia nigra, and hippocampus. EPA-DHA-loaded nanosponges increased hippocampal SOD and catalase activity by approximately 60% [191].

Collectively, these findings underscore that nanocarrier-based strategies are critical to maximize CNS delivery, bioavailability, and therapeutic efficacy of natural compounds, supporting their potential clinical application in NDs.

## 8. Conclusions

Targeting the multifactorial neurotoxic processes underlying NDs remains a major therapeutic challenge. Natural bioactive compounds are promising candidates due to their ability to modulate the Nrf2/ARE signaling cascade, a central hub coordinating redox balance, inflammation, mitochondrial function, and protein quality control.

Preclinical evidence links Nrf2 activation to the regulation of proteostasis through transcriptional control of proteasome activity, autophagy, and endoplasmic reticulum stress responses. These mechanisms may contribute—directly or indirectly—to the clearance of misfolded and aggregation-prone proteins particularly under conditions of oxidative and proteotoxic stress.

Although many natural compounds derived from plants, medicinal fungi, and marine sources attenuate oxidative stress and neuroinflammation and influence protein homeostasis, direct evidence that their anti-aggregatory effects are exclusively Nrf2-mediated remains limited. Likely, Nrf2-dependent redox control cooperates with Nrf2-independent mechanisms, such as direct interference with aggregation, modulation of pathological phosphorylation, and mitochondrial stabilization, to produce neuroprotection.

Despite encouraging preclinical results, clinical translation remains limited by poor oral bioavailability, low solubility, extensive metabolism, and limited BBB penetration, all of which reduce systemic and CNS exposure. Advances in formulation strategies may help to overcome these hurdles and support translation into clinical practice.

Currently available pharmacological therapies for NDs remain largely symptomatic and insufficiently address the core molecular drivers of neurodegeneration, including oxidative stress and protein aggregation. This therapeutic gap underscores the relevance of exploring natural Nrf2 modulators, which may act as complementary or, in some cases, synergistic agents alongside conventional therapies. Developing optimized formulations and conducting rigorous clinical studies will be essential to fully realize their translational potential and maximize neuroprotective effects.

## Figures and Tables

**Table 1:** Direct and indirect contributions of Nrf2 to the clearance of misfolded proteins.

Nrf2 Contribution	Mechanism/Targets	Functional Outcome/Examples
Direct (transcriptional control)	Upregulation of autophagy-related genes (p62/SQSTM1, LC3)	Facilitates clearance of misfolded and aggregation-prone proteins (e.g., α-syn, Aβ, tau); enhances proteostasis and proper protein folding/degradation
Indirect (cellular environment modulation)	Reduction in ROS levels	Maintains proteome stability; prevents protein misfolding; preserves autophagic flux
	Anti-inflammatory signaling	Reduces protein aggregation indirectly by limiting inflammation-mediated damage
	Activation of chaperone response (HSF1, HSPs)	Supports protein folding and stress response
	Maintenance of autophagy/proteasome efficiency	Preserves protein clearance capacity
	Modulation of cellular signaling pathways (AMPK, PI3K/Akt, SIRT1, others)	Promotes an environment favorable for protein clearance

Abbreviations: Aβ, amyloid-β; AMPK, AMP-activated protein kinase; α-syn, α-synuclein; HSF1, heat shock factor 1; HSPs, heat shock proteins; LC3, microtubule-associated protein 1 light chain 3; Nrf2, nuclear factor erythroid 2-related factor 2; PI3K, phosphoinositide 3-kinase; ROS, reactive oxygen species; SIRT1, sirtuin 1; p62/SQSTM1, sequestosome 1; tau, microtubule-associated protein tau.

**Table 2:** Major plant-derived phenolic compounds with reported Nrf2-related neuroprotective and proteostasis-modulating effects.

Phenolic Compounds	Phytochemicals (Examples)	Mechanism of Nrf2 Activation	Nrf2 Involvement in Neuroprotection	Proteostasis/Protein Misfolding
Phenolic acids	Gallic acid	Indirect ^1^	↓ ROS-induced neuronal damage; ↑ antioxidant and cytoprotective enzymes; ↓ proinflammatory cytokines	↓ protein oxidation (Nrf2-mediated)
	Ferulic acid, caffeic acid	Mixed ^2^	Contributes to redox homeostasis and cytoprotective responses	Destabilizes Aβ fibrils; prevents new aggregate formation; ↓ protein oxidation (Nrf2-mediated)
Flavonoids	Quercetin	Indirect ^3^ (or mixed ^2^)	↓ oxidative stress-induced neuronal damage; ↑ antioxidant defenses	Covalent binding to Aβ and α-syn; ↑ molecular chaperon expression (partially Nrf2-mediated); ↓ protein oxidation (Nrf2-mediated)
	Baicalin	Indirect ^3^	↓ neuroinflammation; ↑ antioxidant and cytoprotective enzymes	Interacts with protein fibrils; ↓ protein oxidation (Nrf2-mediated)
	Genistein, daidzein, glycitein	Indirect ^4^	↑ antioxidant and cytoprotective enzymes; ↓ lipid and protein oxidation	Binds Aβ monomers, oligomers, and fibrils; activates autophagic pathways; indirect effects via redox control (Nrf2-mediated)
Non-flavonoids (polyphenolic)	Resveratrol	Indirect ^5^	↓ ROS, neuroinflammation, and mitochondrial dysfunction; ↑ phase II enzymes	↑ autophagic clearance; ↓ protein oxidation (partially Nrf2-mediated)
	Lignans (e.g., styraxlignolide A, egonol)	Indirect ^4^	↑ antioxidant defenses; ↓ neuroinflammation	Prevent Aβ_42_ aggregation; ↓ protein oxidation (partially Nrf2-mediated)
Diarylheptanoids (phenolic)	Curcumin	Direct ^6^; minor indirect ^3^	↓ inflammation, oxidative stress, and aggregation-prone environments; restores autophagy via p62-Keap1-Nrf2 pathway; ↑ phase II detoxification and antioxidant enzymes	↓ GSK3β; modulates Aβ aggregation (enriches soluble oligomers/prefibrillar species with reduced neurotoxicity); enhances clearance of misfolded α-syn via LC3-II upregulation (partially Nrf2-dependent)

^1^ Indirect: via redox modulation; ^2^ Mixed: partial direct (Keap1 cysteine modification) + indirect (MAPK, redox signaling); ^3^ Indirect: redox signaling, kinase pathways; ^4^ Indirect; kinase pathway (e.g., PI3K/Akt, MAPK); ^5^ Indirect: SIRT1/AMPK-mediated; ^6^ Direct: Keap1cysteine modification; ↑: increase; ↓: decrease. Note: Reported mechanisms derive mainly from preclinical studies; the relative contribution of Nrf2-dependent versus Nrf2-independent pathways may vary among compounds and experimental models.

**Table 3:** Randomized clinical trials investigating natural compounds in older adults and patients with NDs.

Natural Compound	Population	Treatment (Duration and Dose)	Main Outcomes vs. Placebo
Quercetin	Older adults (N = 70), 60–79 years	24 weeks, 50 mg/day	Prevention of cognitive decline; improvement in depressive symptoms and motivation
Quercetin glycoside	Older adults (N = 80), 60–75 years	40 weeks, 100 mg/day	Reduced Aβ accumulation; improved reaction time and cerebral blood flow parameters; no significant cognitive improvement
Soy isoflavones	AD (N = 59), >60 years	6 months, 100 mg/day	No improvement in cognitive performance (verbal/visuospatial memory, language, executive and visuomotor functions)
Resveratrol	Mild-to-moderate AD (N = 119), mean age: 71.4 years	52 weeks, 500–2000 mg/day ^1^	Stabilization of CSF and plasma Aβ_40_, Aβ_42_, total tau and p-tau
	Mild-to-moderate AD (N = 56)	56 weeks, 500–2000 mg/day ^1^	Reduced CSF TREM2, MMP-9 and cathepsin D; no changes in MMSE, CDR, ADAS-Cog or NPI
	HD (N = 102), ≥18 years	12 months, 80 mg/day	No data available
Grape-derived polyphenols	MCI (N = 10), 72.2 ± 4.7 years	6 months, 72 g/day	Stable brain metabolism without measurable cognitive gains; metabolic activity in specific cortical regions correlated with better attention and working memory
Resveratrol + Curcumin ^2^	ALS (N = 60), ≥18 years	2 months, 75 mg/day (Resveratrol) + 100 mg/day (Curcumin)	No data available
Curcumin + *Ginko biloba*	AD (N = 30), ≥50 years	1 or 4 g/day (Curcumin) + 120 mg/day (Ginkgo extract)	No improvement in MMSE or Aβ levels; increased plasma vitamin E
Curcumin nanocurcumin	ALS (N = 54), 18–85 years	12 months, 80 mg/day (add-on to riluzole)	Improved survival probability; no effect on ALSFRS-R or muscle strength
Curcumin (multi-compound formulation) ^3^	ALS (N = 42), 62.41 ± 11.05 years	6 months, 600 mg/day (add-on to riluzole)	Modest attenuation of ALSFRS-R decline; improved aerobic metabolism and oxidative stress markers
	ALS (N = 22), 64.1 ± 12.9 years	6 months, 1500 mg/day (add-on to riluzole)	Reduced protein oxidation and IL-6; stabilization of TNF-α; increased antioxidant capacity
*Hericium erinaceus*	MCI (N = 30), 50–80 years	16 weeks, 720 mg/day	Improved HDS-R scores; effects reversed after 4-week washout
	MCI (N = 31), >50 years	12 weeks, 3.2 g/day	Improved MMSE scores
*Hericium erinaceus* enriched with Erinacine A	Mild AD (N = 49), >70 years	49 weeks, three times daily, 350 mg/capsule (*H. erinaceus*) + 5 mg/g (erinacine A)	Improved MMSE and IADL; stabilization of calcium, albumin, ApoE and Aβ_1–40_
DHA/EPA	Moderate AD (N = 50)	6 months, 430 mg DHA + 150 mg EPA/day	Immunomodulatory effects without significant changes in oxidative stress markers
ω-3 PUFAs (flaxseed oil) + vitamin E	PD patients (N = 40)	12 weeks, 1000 mg/day (ω-3 PUFAs) + 400 IU/day vitamin E	Significant improvement in UPDRS; increased TAC and GSH

^1^ Dose escalation: 500 mg increments every 13 weeks; ^2^ liposomal formulations; ^3^ lactoferrin-based carrier; Abbreviations: AD, Alzheimer’s disease; ALS, amyotrophic lateral sclerosis; ALSFRS-R, ALS functional rating scale–Revised; ApoE, apolipoprotein E; Aβ, amyloid-β; CSF, cerebrospinal fluid; DHA, docosahexaenoic acid; EPA, eicosapentaenoic acid; GSH, glutathione; HD, Huntington’s disease; HDS-R, hasegawa dementia scale–revised; IADL, instrumental activities of daily living; IL-6, interleukin-6; MCI, mild cognitive impairment; MMSE, mini-mental state examination; NPI, neuropsychiatric inventory; PD, Parkinson’s disease; p-tau, phosphorylated tau; TAC, total antioxidant capacity; TNF-α, tumor necrosis factor-α; UPDRS, unified Parkinson’s disease rating scale; ω-3 PUFAs, omega-3 polyunsaturated fatty acids.

## Data Availability

No new data were created or analyzed in this study. Data sharing is not applicable to this article.

## Abbreviations

The following abbreviations are used in this manuscript:

3-NP	3-Nitropropionic acid
4-HNE	4-hydroxynonenal
8-oxodG	8-oxo-7,8-diidro-2′-deossiguanosina
AD	Alzheimer diseases
ADAS	Alzheimer’s disease assessment scale
ALS	Amyotrophic lateral sclerosis
ALSFRS-R	ALS Functional Rating Scale-Revised
APP/PS	amyloid precursor protein/presenilin
AREs	antioxidant response elements
Aβ	Amyloid-β
BBB	Blood–brain barrier
BDNF	Brain-Derived Neurotrophic Facto
C9ORF72	chromosome 9 open reading frame 72
CAPE	Caffeic acid phenethyl ester
CDR	Component Resolved Diagnostics
CNS	Central nervous system
DHA	Docosahexaenoic acid
EPA	Eicosapentaenoic acid
ERK	Extracellular signal-regulated kinase
FA	Ferulic acid
FUS	fused in sarcoma
GA	gallic acid
GDNF	Glial Cell Line-Derived Neurotrophic Factor
GPx	glutathione peroxidase
GR	Glutathione reductase
GSH	reduced glutathione
GSK-3β	Glycogen synthase kinase 3 beta
GSPEs	Grape seed polyphenolic extracts
GST	Glutathione S transferase
HACE1	HECT domain and ankyrin repeat-containing E3 ubiquitin protein ligase 1
H_2_O_2_	Hydrogen peroxide
HD	Huntington disease
HE-ETH	Ethanolic basidiocarp extracts
HO-1	Heme-oxygenase 1
HSF1	Heat shock factor 1
HSP	Heat shock protein
IADL	Instrumental activities of daily living
IL	Interleukin
Keap1	Kelch-like ECH-associated protein 1
LC3	Microtubule-associated protein 1 light chain 3
LPS	Lipopolysaccharide
MAPK	Mitogen-activated protein kinase
MCI	mild cognitive impairment
MDA	Malondialdehyde
mHTT	Mutated huntingtin
MMSE	Mini Mental State Examination
MPP^+^/MPTP	1-methyl-4-phenylpyridinium/1-methyl-4-phenyl-1,2,3,6-tetrahydropyridine
NDP52	Nuclear dot protein
NDs	Neurodegenerative diseases
NF-κB	nuclear factor-kappa B
NPI	Neuropsychiatric Inventory
NQO1	NAD(P)H: quinone oxidoreductase 1
Nrf2/Nfe2l2	Nuclear factor erythroid 2–related factor 2
PD	Parkinson disease
PI3K/Akt	Phosphatidylinositol 3-kinase/protein kinase B
PLGA	Poly (lactic-co-glycolic acid)
p-tau	Phosphorylated tau
ROS	Reactive oxygen species
Rbx1	Ring-Box 1
SLN	Solid lipid nanoparticles
SOD	Superoxide dismutase
SPs	Senile plaques
SQSTM1	Sequestosome 1
TAC	Total antioxidant capacity
TBAR	Thiobarbituric acid reactive substances
tBHQ	Tert-butylhydroquinone
TDP-43	TAR DNA-binding protein 43
TNF-α	Tumor necrosis factor-alpha
TREM2	Triggering receptor expressed on myeloid cells 2
UPS	Ubiquitin-proteasome system
α-syn	α-synuclein
β-secretase	BACE1
ω-3 PUFAs	ω-3 polyunsaturated fatty acids
